# No evidence of social learning in a socially roosting butterfly in an associative learning task

**DOI:** 10.1098/rsbl.2022.0490

**Published:** 2023-05-17

**Authors:** Priscila A. Moura, Marcio Z. Cardoso, Stephen H. Montgomery

**Affiliations:** ^1^ Departamento de Ecologia, Universidade Federal do Rio Grande do Norte, Natal, RN, 59078-970, Brazil; ^2^ Departamento de Ecologia, Instituto de Biologia, Universidade Federal do Rio de Janeiro, Rio de Janeiro, RJ, 21941-902, Brazil; ^3^ School of Biological Sciences, University of Bristol, Bristol BS8 1QU, UK

**Keywords:** *Heliconius*, pollen feeding, foraging decision, social information, cognitive ecology, Lepidoptera

## Abstract

Insects may acquire social information by active communication and through inadvertent social cues. In a foraging setting, the latter may indicate the presence and quality of resources. Although social learning in foraging contexts is prevalent in eusocial species, this behaviour has been hypothesized to also exist between conspecifics in non-social species with sophisticated behaviours, including *Heliconius* butterflies. *Heliconius* are the only butterfly genus with active pollen feeding, a dietary innovation associated with a specialized, spatially faithful foraging behaviour known as trap-lining. Long-standing hypotheses suggest that *Heliconius* may acquire trap-line information by following experienced individuals. Indeed, *Heliconius* often aggregate in social roosts, which could act as ‘information centres’, and present conspecific following behaviour, enhancing opportunities for social learning. Here, we provide a direct test of social learning ability in *Heliconius* using an associative learning task in which naive individuals completed a colour preference test in the presence of demonstrators trained to feed randomly or with a strong colour preference. We found no evidence that *Heliconius erato*, which roost socially, used social information in this task. Combined with existing field studies, our results add to data which contradict the hypothesized role of social learning in *Heliconius* foraging behaviour.

## 1. Introduction

Learning to find food, mates, and to avoid danger by observing others is often advantageous in vertebrate societies [1–3]. Social learning can also be achieved by organisms with less expansive nervous systems, such as insects [4–19]. In insects, social learning has been observed when individuals actively pass on acquired information in eusocial species, such as the waggle dance in honeybees [4–6] and the tandem-running recruitment system in ants [7]. It has also been reported as a by-product of copying an animal's behaviour, known as inadvertent social information [8], and is chiefly obtained in contexts of foraging [9–13], mate [14] and oviposition choices [15–17], and predation avoidance [18,19].

When foraging, inadvertent social information via location, visual and chemosensory cues [20] may indicate the presence and quality of resources [8]. Observer bumblebees, for example, use location cues when feeding sites are unfamiliar [9,21]. They also switch flower preference more easily in the presence of a conspecific demonstrator [10,12] and develop a preference for a flower colour after observing a demonstrator [11]. Thus, location and visual cues may increase the probability that the observer will be attracted to a particular location (local enhancement) or to a specific object or colour (stimulus enhancement) [3,11,12].

Although social learning in foraging contexts is more likely in more social species [22,23], it does occur outside these contexts. For example, in *Gryllus bimaculatus*, a subsocial cricket with relatively solitary lives but with sophisticated communication among conspecifics, naive individuals prefer the odour of drinking stations which a demonstrator previously occupied [24]. *Heliconius* butterflies, have also long been hypothesized to use social information [25] in the context of a novel foraging specialization [26–28]. *Heliconius* are the only butterflies to actively collect and digest pollen [29–31], a behaviour associated with increased longevity [32] and delayed reproductive senescence [33], but one that requires specialized foraging behaviours, known as trap-lining, to learn the location of pollen resources [27,32,34,35]. Trap-lining involves learning spatially and temporally faithful foraging routes and provides an efficient strategy for repeated visitation of reliable resources [32,34]. Many *Heliconius* exhibit nocturnal gregarious roosting, with butterflies returning to the same sites from dusk to dawn, from which they start their trap-lines [25–28]. *Heliconius* also have overlapping generations which is suggested to enhance opportunities for social learning [22].

Consequently, social learning has been hypothesized to be important for *Heliconius* in the context of information-sharing of food sources via nocturnal gregarious roosting sites [26,34], which may function as ‘information centres’ [36]. According to this view, new roost mates learn the location of food sources by following experienced individuals [26,34]. However, a key prediction of the information-sharing hypothesis, that younger individuals should follow more experienced individuals out of the roost, was not supported in field experiments, with only one of 256 recorded foraging bouts involving a roost mate following another individual from the roost site to a food source [37]. Furthermore, roost mates have only partially overlapping home ranges and visit different floral resources [27]. Instead, data support the adaptive value of gregarious roosting as enhancing aposematic signals [37]. Nevertheless, following behaviour occurs regularly in butterflies from neighbouring roost sites between food sources [26,28,38], suggesting that opportunities for social learning may still occur.

In this study, we provide the first experimental assessment of social learning ability in *Heliconius.* We focus on *H. erato*, a socially roosting species [39] that has been the focus of field studies on gregarious roosting and social following, as these behaviours were previously believed to act as mechanisms of information transfer [36]. While social learning of foraging routes has been investigated in a small number of species, typically ants [7], the less stereotyped behaviour of *Heliconius*, combined with the complexity and scale of their native habitats, makes this approach more challenging in these butterflies. We therefore adapted well-established associative learning protocols [40], focused on colour preference assays, to assess whether naive butterflies that acted as observers would adjust their initial preference, or more rapidly learn a foraging task, when exposed to older, experienced individuals.

## 2. Materials and methods

### (a) Experimental subjects and arena

Experimental subjects originated from first-generation insectary-reared stock populations of *Heliconius erato phyllis*, descended from multiple wild-caught females collected in Mata do Jiqui, Natal, Brazil (5°55'39″S, 35°10'59″W). We maintained stock populations in outdoor cages (3 × 3 × 2.5 m) in which free-flying butterflies were able to engage in natural social and flight behaviours, including chasing, mating and following. At night, individuals were observed to form roosts of 2–22 individuals. Stock butterflies had access to host plants (*Passiflora misera* and *P. galbana*) and rewarding artificial white flowers. All butterflies were individually labelled with unique IDs. The test arena was composed of purple and yellow artificial flowers. These colours were chosen given that they are, on average, both relatively unpreferred [41]. Rewarding flowers contained an approximately 20% sugar solution mixed with bee-pollen supplement while unrewarding flowers were empty. Twelve flowers of each colour were placed on a grid of 24, with randomized positions (electronic supplementary material, figure S1).

### (b) Selection of demonstrators

**Figure 1.**
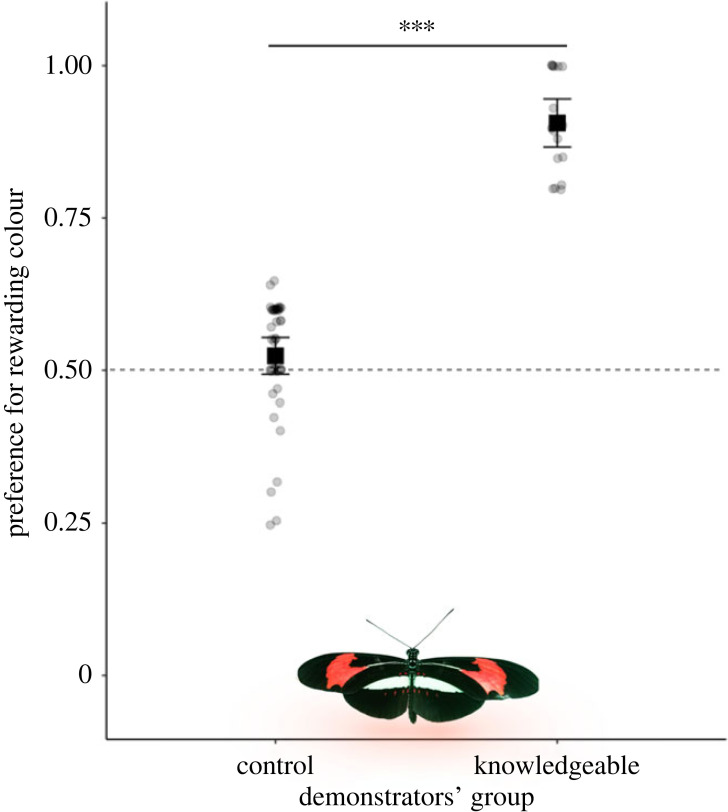
Data from colour preference tests of *H. erato* demonstrators from control and knowledgeable groups following training. Squares and whiskers are means of individuals' preference ± 95% CI. ***p < 0.001.

Butterflies were randomly assigned to one of two demonstrator groups, each collectively subjected to 4 days of training (electronic supplementary material, figure S2). For one group, only purple flowers were positively reinforced to strengthen preference for purple. For the other, the colour of the rewarding flower was randomly determined for each trial. Training was run between 08.00 and 16.00. In the following day, a final 5 min test was conducted to determine demonstrators' colour preferences. Preference was calculated as the proportion of landings on purple and yellow flowers out of 20 landing events. Demonstrators with a less than or equal to 60% purple preference were assigned to the control group, whereas demonstrators with a greater than or equal to 80% purple preference were assigned to the knowledgeable group, creating two demonstrator groups with non-overlapping, unbiased and biased preferences, respectively (figure 1)

### (c) Social learning experiment

The social learning experiment consisted of two phases (electronic supplementary material, figure S3). (i) During *pre-training*, naive butterflies, hereafter ‘observers’, fed on artificial white flowers between 08.00 and 16.00 to get accustomed to artificial flowers. (ii) In the *trialling phase*, which lasted for 4 days, butterflies were randomly assigned to either the control or the knowledgeable demonstrator groups. Observers were released in pairs along with 10 demonstrators to ensure that most of individual-level feeding attempts were by the demonstrators. During trials, we scored the number of feeding attempts made by each observer on purple rewarding flowers and yellow unrewarding flowers for 15 min. A choice was scored when the butterfly landed on a flower.

### (d) Statistical analysis

Data were analysed using generalized linear mixed models in R using lme4 [42]. First, we asked whether demonstrators from different groups preferred the rewarding colour, using a binomial GLM with response variable ‘preference for rewarding colour’ (proportion of landings on purple flowers) and fixed factor ‘group’ (control and knowledgeable). Then, for observers, we examined whether there were intergroup differences in preference for rewarding colour over time, using a binomial GLMM with response variable ‘preference for rewarding colour’ and fixed factors ‘group’ and ‘trial day’ (1–4), with identity set as a random effect. Finally, we analysed whether observers preferred flowers occupied by demonstrators using a binomial GLMM with response variable ‘local preference’ (0 = no; 1 = yes) and fixed factors ‘group’ and ‘trial day’ (1–4), with identity set as a random effect.

## 3. Results

### (a) Demonstrators differ in colour preference after training

Demonstrators were clearly differentiated based on the strength of their colour preferences, with those assigned to the knowledgeable group preferring purple flowers significantly more than those assigned to the control group (figure 1, *z* = 10.13, d.f. = 57, *n* = 60 individuals, *p* < 0.001). Prior to the social learning experiment, demonstrator groups therefore had different preferences for purple and yellow colours.

### (b) Social context does not alter observers' initial preferences

Most individual-level feeding attempts were made by demonstrators (75.7%). Observer preferences were similar with regard to flower colour in individuals foraging alongside knowledgeable and control butterflies in the first day of the trialling phase (electronic supplementary material, figure S4, *z* = −0.44, d.f. = 27, $\sigma_{i}^{2}=0.08$, *n* = 30, *p* = 0.66). This result shows that observer colour preferences did not match demonstrator preferences with both observer groups preferring purple. Furthermore, observers avoided flowers that were occupied by experienced conspecifics, landing preferentially on unoccupied flowers. This behaviour was observed throughout the trials and did not change over time (*z* = −0.19, d.f. = 126, $\sigma_{i}^{2}=0.59$, *n* = 35, *p* = 0.85).

### (c) Social context does not alter learning rate in observers

**Figure 2.**
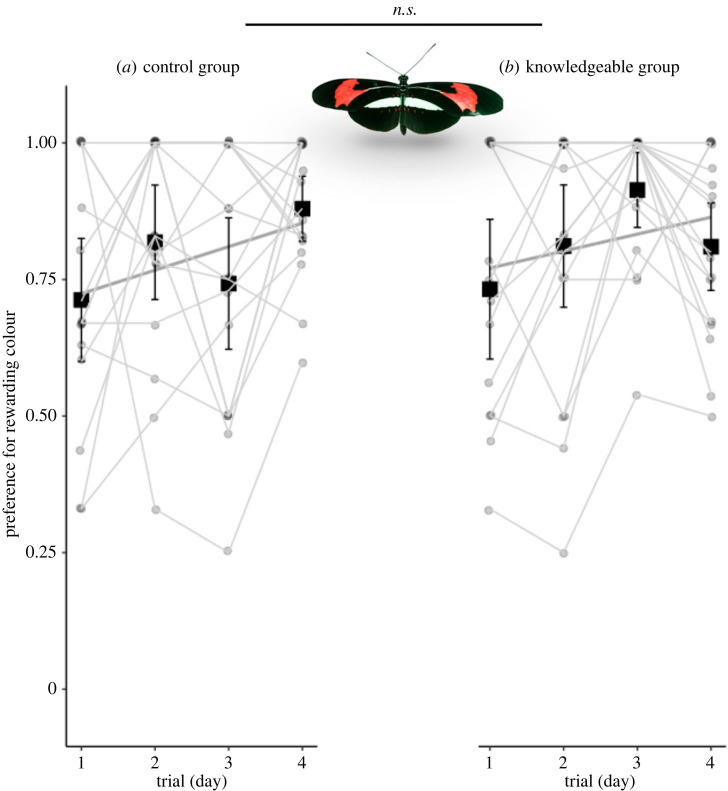
Preference for the rewarding colour in preference trials of *H. erato* observers in (*a*) control and (*b*) knowledgeable groups during the social learning experiment. Points are individual observations. Light grey lines connect the same individual. Squares and whiskers represent means of individuals’ preference ± 95% CI.

Overall, observers’ preferences for the rewarding colour increased over time (*z* = 3.61, d.f. = 127, $\sigma_{i}^{2}=0.46$, *n* = 35, *p* < 0.001, electronic supplementary material, figure S5). However, there was no significant effect of the demonstrator group on colour preference (*z* = 0.50, d.f. = 126, $\sigma_{i}^{2}=0.46$, *n* = 35, *p* = 0.61, figure 2). In fact, observers from the control group tended to show a better overall performance (control: *z* = 3.02, d.f. = 62, $\sigma_{i}^{2}=0.34$, *n* = 17, *p* < 0.01; knowledgeable: *z* = 1.96, d.f. = 62, $\sigma_{i}^{2}=0.59$, *n* = 18, *p* = 0.05). Specifically, 65% of these individuals increased their preference for purple over time, with only 6% showing a decrease, compared to 44% and 22%, respectively, of individuals from the knowledgeable group. It is possible that the high mean naive preference for the positively rewarded colour masks the potential for social learning. To explore this effect, we conducted two analyses: first, we sub-sampled the data to only include individuals with naive preferences outside the range of the knowledgeable demonstrators (less than 70%). We again found no effect of group (*t* = −1.897, d.f. = 12, *n* = 14, *p* = 0.08). Second, we compared the relationship between naive preference and the shift in preference after training (trial 4–trial 1). We predicted that if individuals with weaker preferences more readily learn social information, we would expect this relationship to differ between the two treatment groups. This prediction was not met (*t* = −0.761, d.f. = 12, *n* = 14, *p* = 0.46).

## 4. Discussion

Using a classic paradigm for learning through observation, we found no evidence that *Heliconius erato* feeding preferences are influenced by social cues. Neither the colour preference nor learning rate of naive butterflies was affected by the behaviour of experienced conspecifics that had a strong preference for the rewarding colour. Naive individuals also showed no preference for feeding from resources recently visited by conspecifics. Our study therefore shows that conspecifics are not attracting others to a specific flower colour (stimulus enhancement) or to a particular flower (local enhancement).

Our study was motivated by a long-held hypothesis that naive *Heliconius* butterflies learn foraging routes between pollen resources by social following experienced individuals. Given the difficulty of testing this hypothesis directly, we focused on the general capacity of *H. erato* to use social information in a simpler assay. However, we argue this test has biological relevance for the following reasons: first, in our cages free-flying individuals still engage in social interactions, providing the opportunity for following to the feeders. Second, for social learning of foraging behaviour to occur in the wild, naive individuals must follow experienced individuals to the point where they feed, otherwise there is no obvious reinforcement of the locality cues; as such, our experiment focuses on a key point in this interaction. Finally, floral colour cues do have significance in the wild, with most *Heliconius* collecting pollen from a restricted, but variable range of plant species [43], which may be used in the context of external cues, such as time of day [44]. As such, if *H. erato* pays attention to conspecific foraging behaviours, we argue the current experiment should capture this ability. One potential limitation of our data is the relatively biased naive preference for the rewarded colour. It is possible that this preference renders asocial learning an efficient strategy, with social learning being solely deployed in contexts where asocial learning is ineffective [45]. However, we do not see evidence of this effect in our data, when exploring the interaction between the naive individual preference and the social treatment.

While we cannot formally rule out the possibility that *Heliconius* use social cues in other contexts, we suggest our results support the conclusion that the impact of conspecific behaviour on learned foraging behaviours may be overestimated in *Heliconius* [27,28]. Consistent with this interpretation, previous descriptions of following behaviour suggest this does not regularly lead to feeding resources [38]. Arguably, conspecifics could still play a role by attracting others to a general location, such as a pollen source, and indeed co-roosting individuals can have overlapping foraging routes [27,28]. However, this result can also be explained by a preference for resources in proximity to their roosting sites [27,28] within a relatively small and stable home range [25,27,46], and field data suggest very low levels of conspecific following on leaving the roost [28].

In the absence of social learning, foraging decisions in *Heliconius* butterflies may be influenced by innate biases, and individual experience. If our results generalize, *Heliconius* either do not learn the links between social context and reward or may not need to because social information is less reliable or more costly than direct experience. Of potential relevance, is the generally patchy distribution of pollen resources [34]. Social information is potentially inefficient in this context because of increased competition and depletion of limited resources. Indeed, in our experiment, observer butterflies from the knowledgeable group tended to show a lower learning rate, perhaps due to a competition effect. In this case, knowledgeable demonstrators were more likely to feed on purple flowers expelling the observers, which preferred unoccupied flowers.

Social information is, nevertheless, prevalent in a range of insects with complex foraging needs for which many of the arguments above would also apply. This includes bumblebees, for example, a social species that not only copy feeding techniques of conspecifics but are also able to improve on them [47], suggesting behavioural flexibility [48]. For non-colonial insects, especially aggregating solitary insects, the use of inadvertent social information has been observed in contexts of (i) mate choice, with females exploiting public information to select mates [14]; (ii) oviposition choice, with females preferring to lay eggs on food substrates associated with female conspecifics [15,16] or other social cues [49] and (iii) predator-avoidance behaviours mediated by chemical cues [18] and by the observance of conspecific hiding behaviour [19]. Although, in foraging contexts, social learning has been reported only in a cricket species [24], the use of inadvertent social information is not as widely reported, suggesting it may be less prevalent among foraging solitary insects. Consistent with this interpretation, our experiment provides no evidence of social learning in *H. erato*. This, together with field data contradicting the information centre hypothesis and the hypothesis that following leads to floral resources [27,28], casts doubt on the role of social learning in supporting the specialized foraging behaviour of *Heliconius*.

## Data Availability

Data and R scripts are available from the Dryad Digital Repository: https://doi.org/10.5061/dryad.7h44j0zwz [50]. The data are provided in the electronic supplementary material [51].
